# Interleukin-2 signalling is modulated by a labile disulfide bond in the CD132 chain of its receptor

**DOI:** 10.1098/rsob.110036

**Published:** 2012-01

**Authors:** Clive Metcalfe, Peter Cresswell, A. Neil Barclay

**Affiliations:** 1 Sir William Dunn School of Pathology, University of Oxford, Oxford OX1 3RE, UK; 2 Department of Immunobiology, Howard Hughes Medical Institute, Yale University School of Medicine, 300 Cedar Street, New Haven, CT 06520-8011, USA

**Keywords:** disulfides, membrane proteins, redox, cytokine receptor, CD132, IL-2

## Abstract

Certain disulfide bonds present in leucocyte membrane proteins are labile and can be reduced in inflammation. This can cause structural changes that result in downstream functional effects, for example, in integrin activation. Recent studies have shown that a wide range of membrane proteins have labile disulfide bonds including CD132, the common gamma chain of the receptors for several cytokines including interleukin-2 and interleukin-4 (IL-2 and IL-4). The Cys^183^–Cys^232^ disulfide bond in mouse CD132 is susceptible to reduction by enzymes such as thioredoxin (TRX), gamma interferon-inducible lysosomal thiolreductase and protein disulfide isomerase, which are commonly secreted during immune activation. The Cys^183^–Cys^232^ disulfide bond is also reduced in an *in vivo* lipopolysaccharide (LPS)-induced acute model of inflammation. Conditions that lead to the reduction of the Cys^183^–Cys^232^ disulfide bond in CD132 inhibit proliferation of an IL-2-dependent T cell clone and concomitant inhibition of the STAT-5 signalling pathway. The same reducing conditions had no effect on the proliferation of an IL-2-independent T cell clone, nor did they reduce disulfide bonds in IL-2 itself. We postulate that reduction of the Cys^183^–Cys^232^ disulfide in CD132 inhibits IL-2 binding to the receptor complex. Published data show that the Cys^183^–Cys^232^ disulfide bond is exposed at the surface of CD132 and in close contact with IL-2 and IL-4 in their respective receptor complexes. In addition, mutants in these Cys residues in human CD132 lead to immunodeficiency and loss of IL-2 binding. These results have wider implications for the regulation of cytokine receptors in general, as their activity can be modulated by a ‘redox regulator’ mechanism caused by the changes in the redox environment that occur during inflammation and activation of the immune system.

## 2. Introduction

Disulfide bonds are important in maintaining the structure of extracellular and secreted proteins, but it is now apparent that some disulfide bonds are labile and their reduction leads to functional changes [1,2]. The uptake of some viruses such as HIV [3], Newcastle disease virus [4] and hepatitis C virus [5] is redox-dependent. Platelet activation is associated with redox-induced changes at the cell surface [6,7]. Disulfide bonds in the β2 integrins and αIIbβ3 integrin maintain their low affinity state, and thrombus formation is controlled by the redox state of disulfide bonds in the beta chain of αIIbβ3 [8,9]. The affinity of integrins on other cell types can also be modulated by reducing agents [10]. For example, the α4 chain integrin α4β1 was shown to have free sulfydryl groups on treatment of cells with reducing agents [10,11]. The powerful anti-microbial activity of human β-defensin-1 is revealed after reduction of disulfide bonds [12]. IL-4 has been shown to contain a disulfide bond susceptible to mild reduction [13]. Some ligands for cell surface receptors are also redox sensitive (e.g. MICA) [14]. Immunization increases the number of free thiols in lymph nodes [15]. Activation of T cells by dendritic cells leads to secretion of thioredoxin (TRX) into the extracellular space [16], where it can catalyse reduction of disulfide bonds on the surface of cells with which it comes into contact. This activation and TRX secretion can be limited by the addition of regulatory T cells [17,18]. In a model of arthritis, disease susceptibility was shown to correlate with the level of free thiols at the surface of T cells, and this was dependent on intracellular reactive oxygen levels [19], suggesting a role for redox chemistry in autoimmunity. Combined, these data suggest a central role for redox chemistry at the cell surface in controlling the levels of the immune response to antigen.

We have recently developed a proteomics-based technique to determine the membrane proteins of leucocytes that have labile disulfides using mild reducing conditions such as dilute tris(2-carboxyethyl)phosphine (TCEP), and enzymes such as TRX, gamma interferon-inducible lysosomal thiolreductase (GILT) and protein disulfide isomerase (PDI) that are commonly secreted during immune activation [20,21]. After reduction, the proteins with labile disulfide bonds were labelled with maleimide-PEO_2_-biotin (MPB). This enabled these proteins to be purified by avidin affinity chromatography and, following digestion with trypsin, to be identified by mass spectrometry. Leucocyte membrane proteins with labile disulfide bonds were surprisingly common and included various classes of proteins such as integrins, transporters and adhesion proteins, suggesting the activity of many proteins may be modulated by redox changes during immune responses [20]. One protein identified in these screens was CD132, which is the common signalling component (γ_c_) of the receptors for several cytokines, namely IL-2, IL-4, IL-7, IL-9, IL-15 and IL-21 [22]. IL-2 plays a central role in T cell activation and regulation [23–25], and we now show that reduction of this disulfide in the CD132 chain of its receptor has a marked effect on IL-2 signalling. This suggests that responsiveness to IL-2 (and possibly other cytokines) may be modulated by changes in the redox environment that occur during immune responses.

## 3. Results

### 3.1. Identification of a labile disulfide in CD132

**Figure 1.**
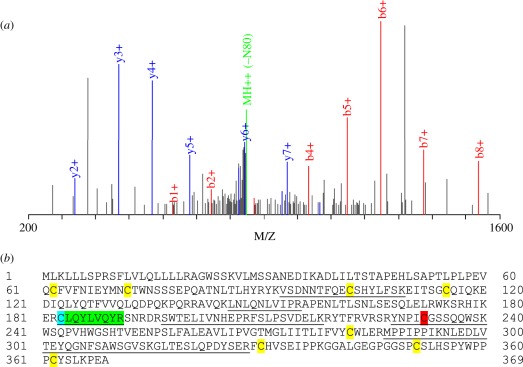
Analysis of CD132 after reduction with PDI showing peptide coverage and MBP-modified peptide. (*a*) The MS/MS spectrum of peptide C(MPB)LOYLVQRR shows good unambiguous coverage of the b+ (red peaks) and y+ (blue peaks) ion series. Sequential individual amino acid masses were identified in both the b+ and y+ ion series. (*b*) Amino acid sequence of mouse CD132 showing the peptides identified by mass spectrometry (underlined) and the peptide containing the biotin-maleimide modification (green; Cys ^183^ in blue), which forms a labile disulfide bond with the Cys^232^ shown in red. Other Cys residues in the structure are shown in yellow.

The CD132 chain was identified as containing a redox-labile disulfide bond (Cys^183^–Cys^232^) in screens of T cells treated with the chemical reductant TCEP and three enzymatic reductants (TRX, GILT and PDI), and also in spleen cells from mice treated with LPS in a short-term model of inflammation [20]. The peptides identified by mass spectrometry are summarized in table 1. The three enzymatic reductants gave similar peptide coverage, which was higher than seen by TCEP reduction, an observation seen for other proteins in the global screen [20]. One Cys residue in CD132 was identified as an MPB-labelled peptide with the sequence C*LQYLVQYR (table 1; the asterisk indicates the modified Cys residue), the MS/MS spectrum for this peptide obtained after PDI reduction (similar spectra were obtained for the other reducing agents) is shown in figure 1*a*. This indicates that this Cys (residue 183) originated from a disulfide bond that was reduced by the mild reducing conditions described above and previously [20]. A peptide containing Cys^232^, the other residue that makes up this disulfide bond, was not detected with an MPB label. However, it was detected in an unmodified carboimidomethyl form. The protein sequence of mCD132 and identified peptides are shown in figure 1*b*. The MPB label reduces the ionization efficiency of the peptide compared with unlabelled carboimidomethyl Cys (C. Metcalfe 2011, unpublished data) containing peptides, thus it is possible that the Cys^232^-containing peptide was undetectable under the conditions employed in the study. Under TRX- and GILT-reducing conditions, the C*LQYLVQYR peptide was detected with both MPB and carbamidomethyl modifications, indicating that modification of both Cys residues of the labile disulfide bond was not 100 per cent efficient. In the *in vivo*, LPS inflammation model CD132 was detected although no MPB modified peptide was detected in CD132 or indeed any other protein [20]. This is due to the complexity of the sample. Peptides originate from proteins of many different cell types and therefore will be present at levels at the limit of detection of our mass spectrometry system. Only a small percentage of these peptides will be labelled with MPB and are therefore unlikely to be identified on probability grounds.

**Table 1.** Summary of CD132 reduction data from TCEP, PDI, TRX and GILT reductase extracted from global screens of a 2B4 T cell clone. Data show the percentage coverage of CD132, the CD132 peptides identified and any modifications indicated with their positions within the identifed peptide. Cys denotes the residue number of the cysteines in the immature mouse CD132. Cys^183^ forms a disulfide with Cys^232^ and the equivalent in the human protein structure is Cys^160^–Cys^209^, and the latter nomenclature is used in the discussion. CD132 was also detected in spleen cells following LPS induction of inflammation.

reducing agent	percentage coverage	peptides	modifications	Cys
TCEP	2.0	CLQYLVQYR	MPB@1	183
PDI	30.9	FSLPSVDELKR		
		LNLQNLVIPR		
		NLEDLVTEYQGNFSAWSGVS		
		SWTELIVNHEPR		
		YNPICGSSQQWSK	carbamidomethyl@5	232
		CLQYLVQYR	MPB@1	183
		GLTESLQPDYSER		
		MPPIPPIK		
		VSDNNTFQECSHYLFSK	carbamidomethyl@10	102
thioredoxin	31.4	FSLPSVDELKR		
		GLTESLQPDYSER		
		LNLQNLVIPR		
		NLEDLVTEYQGNFSAWSGVSK		
		SWTELIVNHEPR		
		YNPICGSSQQWSK	carbamidomethyl@5	232
		CLQYLVQYR	MPB@1	183
		CLQYLVQYR	carbamidomethyl@1	183
		EITSGCQIQK	carbamidomethyl@6	115
		VSDNNTFQECSHYLFSK	carbamidomethyl@10	102
GILT	26.8	FSLPSVDELKR		
		GLTESLQPDYSER		
		LNLQNLVIPR		
		NLEDLVTEYQGNFSAWSGVSK		
		SWTELIVNHEPR		
		CLQYLVQYR	MPB@1	183
		CLQYLVQYR	carbamidomethyl@1	183
		YNPICGSSQQWSK	carbamidomethyl@5	232
		EITSGCQIQK	carbamidomethyl@6	115
LPS	6.8	SWTELIVNHEPR		
		GLTESLQPDYSER

### 3.2. The labile disulfide bond in CD132 is exposed to solvent

**Figure 2.**
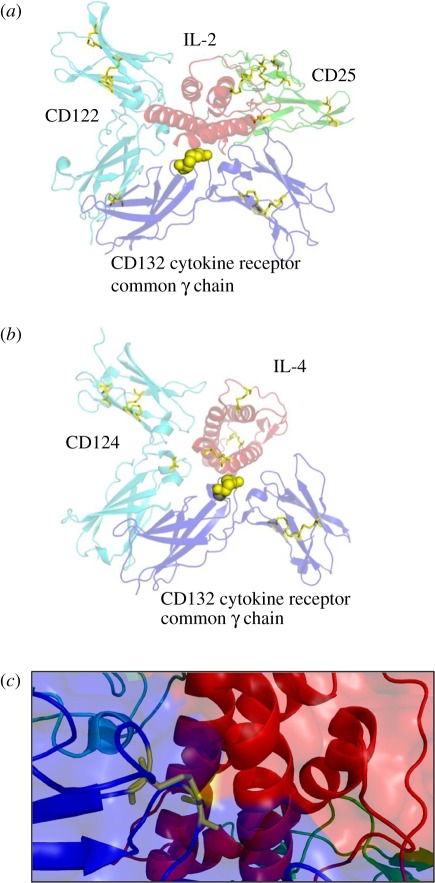
Structures of (*a*) the human IL-2 receptor complex (PDB accession 2ERJ) and (*b*) the IL-4 receptor complex (PDB accession 3BPL) in complex with IL-2 and IL-4, respectively. CD132 is shown in mauve, the cytokine (IL-2 and IL-4) in brown, the beta chain of the receptor in blue. The IL-2 receptor complex has an additional chain, CD25, shown in green. The Cys^160^–Cys^209^ disulfide bond identified as being labile in mouse CD132 corresponds to the same numbered residues in the human structure and is shown as yellow spheres and lies in contact with the cytokine in both complexes. The other non-labile disulfide bonds are represented as yellow sticks. (*c*) Close up of the Cys^160^–Cys^209^ disulfide bond (yellow sticks) on CD132 (blue cartoon and surface) showing that is bridging two loops that are in direct contact with IL-2 (red cartoon and surface).

The structure of the CD132 ectodomain has been determined in complexes of both the human IL-2 and IL-4 receptors with IL-2 and IL-4, respectively (reviewed by LaPorte *et al*. [26]). CD132 contains two fibronectin type III domains. The peptide containing the MBP modified Cys identified in mouse CD132 (see above) corresponds to Cys^160^ that forms a disulfide bond with Cys^209^ in these structures for human CD132 (figure 2). This domain type has a fold similar to an Ig-like domain consisting of two beta sheets but it usually lacks disulfide bonds in the typical Ig inter-sheet position. However, in some cases disulfide bonds are present, such as the Cys^160^–Cys^209^ disulfide of CD132. This disulfide bond is unusual in that it is at the surface of the protein (figure 2*a*) with a large solvent accessibility area of 56 Å^2^ (compared with a structure average of 19 Å^2^) when calculated in the absence of IL-2, which is presumably the state under which redox regulation would occur. This Cys^160^–Cys^209^ disulfide bond is relatively short, with a Cα–Cα of 5.3 Å compared with an average of 5.9 Å for the other disulfide bonds present in the IL-2 and IL-4 receptor structures. These features are compatible with the possibility that this disulfide bond might be readily reduced. A recent bioinformatics study that analysed all of the disulfide bonds in the protein data bank based on solvent accessibility, Cα–Cα distance and an estimation of torsion strain on the S–S bonds [1,2] concluded that the most common configuration of the known allosteric disulfide bonds is the –RHStaple. A feature of –RHStaple bonds is the close proximity of the α-carbon atoms of the two cysteine residues [27,28]. In both of these structures, the Cys^160^–Cys^209^ disulfide bond sits at the interface of CD132, linking two beta loops as shown in figure 2*c* with the cytokine in direct contact with the helix bundle. The possibility that mild reducing conditions typical of those found in T cell activation might reduce this disulfide bond enough to affect the ability of IL-2 to bind and signal through the receptor was investigated.

### 3.3. Reducing agents inhibit the proliferation of an IL-2-dependent cell line

**Figure 3.**
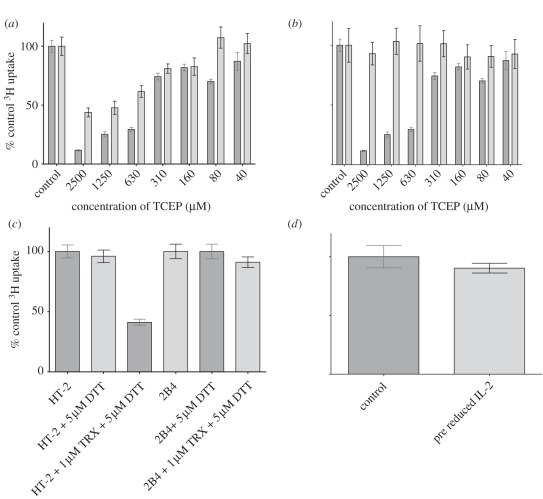
Mild reducing conditions cause inhibition of proliferation of an IL-2-dependent cell line HT-2. (*a*) Mild reduction with TCEP gives inhibition of proliferation of HT-2 cells determined by ^3^H-thymidine incorporation (normalized to 100% for no treatment). The effect is largely reversible as washing restores proliferation levels. Dark grey bars, HT-2 non-washed; light grey bars, HT-2 washed. (*b*) Similar reducing conditions give no inhibition of proliferation of the 2B4 T cell hybridoma (IL-2-independent) compared with the unwashed sample in (*a*). Dark grey bars, HT-2; light grey bars, 2B4. (*c*) The reducing enzyme thioredoxin (TRX) causes inhibition of proliferation of HT-2 cells, but not the 2B4 T cell hybridoma (5 µM DTT is present as an electron donor for TRX enzymatic turnover). (*d*) Treatment of purified IL-2 with 2.5 mM TCEP gives no significant difference in its ability to permit proliferation of HT-2 cells. Error bars indicate standard deviation from three independent experiments.

Some T cell lines are dependent on IL-2 for growth and are widely used as bioassays for IL-2. From the above, we predict that the growth of such a line would be inhibited by mild reducing conditions by affecting the ability of CD132 to signal in response to IL-2. One commonly used line is the T helper clone HT-2 cell line that had been derived from mouse Balb/c spleen cells stimulated with sheep erythrocytes and grown in IL-2 [29]. This line was treated with TCEP and its effects on proliferation were determined by incorporation of ^3^H-thymidine. Proliferation was inhibited in a dose-dependent manner at concentrations of TCEP comparable to those used to reduce the labile disulfide bond in CD132 (figure 3*a*). Washing the TCEP from the cells gave recovery of proliferation to approximately 80 per cent of control levels indicating that inhibition was reversible on a timescale of hours and TCEP caused no significant permanent toxicity to the cell line. In contrast, the same reducing conditions had no effect on the proliferation of the 2B4 hybridoma T cell line that is not dependent on exogenous IL-2 for proliferation, but still expresses CD132 (figure 3*b*).

In order to mimic physiological conditions more closely than TCEP, the effects of TRX were tested. TRX is an enzyme that reduces disulfide bonds to the free sulfydryls and is known to be produced by dendritic cells and required for T cell activation [16]. TRX (in the presence of trace levels of 5 μM dithiothreitol, DTT, as an electron donor) gave similar inhibition of the HT-2 cells as TCEP (figure 3*c*). The TRX had no effect on proliferation of the 2B4 cell line. These results are consistent with previous data showing mutation of this disulfide bond in CD132 inhibits IL-2 binding and signalling [30]. In combination, these results show the Cys^160^–Cys^209^ disulfide bond is redox-labile and that CD132-dependent cytokine binding can be affected by changes in redox potential at the cell surface.

### 3.4. The disulfide bond in IL-2 is resistant to mild reduction

**Figure 4.**
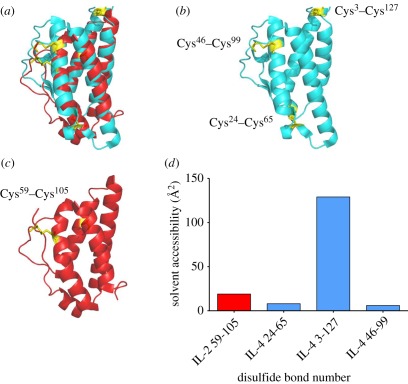
The structures of IL-2 and IL-4 are similar, but one disulfide bond in IL-4 is massively exposed to solvent. (*a*) Superposition of human IL-2 (red) and human IL-4 (green), derived from structures of the whole IL-2 and IL-4 cytokine receptor complex PDB ID:2ERJ and 3BPL, respectively, showing the similarity of the structures. (*b*) Structure of IL-4 alone showing the position of the three disulfide bonds (yellow sticks). (*c*) Structure of IL-2 alone showing the position of its disulfide bond (yellow sticks) and free cysteine (yellow). (*d*) Solvent accesibility of the disulfide bonds in IL-2 (red bars) and IL-4 (blue bars). Calculated using software [31] and http://www.cancerresearch.unsw.edu.au/CRCWeb.nsf/page/Disulfide+Bond+Analysis.

Reduction of the cytokine IL-4 by exogenous reducing agents has been shown to inhibit its ability to bind to the IL-4 receptor complex and inhibits proliferation of an IL-4-dependent cell line [13]. A similar reduction of IL-2 could account for, or at least contribute to, the effects seen on the HT-2 cell line. To test this, recombinant rat IL-2 was incubated under the same reducing conditions known to give extensive inhibition of HT-2 cell proliferation. N-ethylmaleimide (NEM) was added to modify any free sulfydryl residues generated. The IL-2 protein was denatured with urea, fully reduced with TCEP and alkylated with iodoacetamide (IAA). The protein was digested with trypsin and peptides analysed by mass spectrometry. The Cys-containing peptides were analysed for differential labelling with the two thiol-reactive agents using a simple semi-quantitative analysis based on the total ion counts of the peptides' precursor scan peaks. This showed that for the FECQFDDEPATVVEFLR peptide less than 1 per cent contained the NEM-modified Cys compared with the IAA-modified Cys (non-reduced in the native protein) and for the HLQCLENELGALQR peptide about 28 per cent was in the NEM-modified form (table 2). These data were almost identical to a control that had not been reduced. The NEM modification observed was probably due to some denatured protein in the IL-2 preparation. This indicates that the disulfide bond in IL-2 was not labile. IL-2 and IL-4 contain one and three disulfide bonds, respectively, and these disulfide bonds are conserved across several species. X-ray crystal structures are not available for rat or mouse IL-2 or IL-4, but human structures are available for both (figure 4). The tertiary structures of human IL-2 and IL-4 are very similar (figure 4*a*), with good overlay of the 4-helix bundle. Both structures contain a disulfide bond between the second helix of the bundle and an external loop (figure 4*b*,*c*). IL-4 contains two further disulfide bonds (figure 4*b*) and IL-2 contains a conserved ‘free’ cysteine buried inside of the 4-helix bundle (figure 4*c*). Figure 4*d* shows the calculated solvent accessibility for all of the disulfide bonds in IL-2 and IL-4. The Cys^59^–Cys^105^ disulfide bond in IL-2 is buried, as are the Cys^24^–Cys^65^ and Cys^46^–Cys^99^ disulfide bonds in IL-4. However, the Cys^3^–Cys^127^ disulfide bond in IL-4 is highly solvent exposed and is likely to be the one that is redox-labile in the previous study [13].

**Table 2.** Precursor ion intensities of Cys-containing peptides for trypsin digest of TCEP-reduced recombinant rat IL-2. Each peptide was observed multiple times owing to deaminidation. The figures given are for the sum of the ion intensities for each set of peptides either non-reduced (carboimidomethyl modification) or reduced (NEM modification). Ratios of precursor ion intensities were used to determine the relative level of reduction of the disulfide bond in IL-2. There are no structures for rat or mouse IL-2, but the Cys residues in rat IL-2 (78 and 126 indicated after the modification; UNIPROT accession; P17108) correspond to residues 58 and 105 in the X-ray crystal structure of human IL-2 (figure 2 and PDB code 2erj).

peptide	modification	sum of precursor ion intensities
HLQCLENELGALQR	carbamidomethyl@4 [78]	13 100 000
HLQCLENELGALQR	N-ethylmaleimide@4 [78]	5 200 000
FECQFDDEPATVVEFLR	carbamidomethyl@3 [126]	29 600 000
FECQFDDEPATVVEFLR	N-ethylmaleimide@3 [126]	184 000

Pre-reduced and alkylated recombinant rat IL-2 was just as effective as normal IL-2 in controlling the proliferation of the HT-2 IL-2-dependent cell line (figure 3*d*). Therefore, there is no evidence that the effect of reducing agent on the IL-2-dependent cell line is due to effects on the IL-2 itself.

### 3.5. Reduction of CD132 inhibits signalling through the Jak-3–STAT-5 pathway

**Figure 5.**
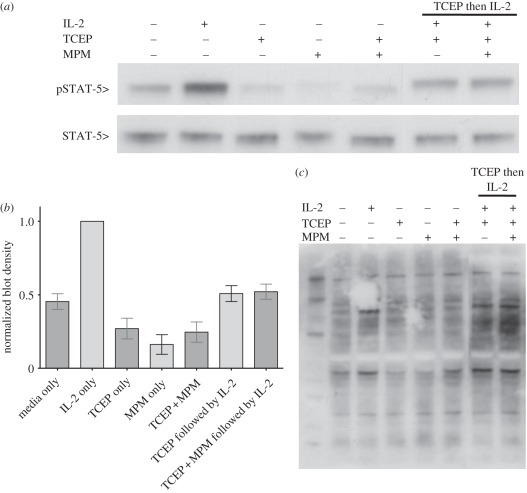
Reduction of CD132 inhibits cell proliferation through the STAT-5 pathway. (*a*) Lysates from HT-2 cells treated with combinations of IL-2, TCEP and MPM (to block free Cys residues) were probed by Western blot with anti-phospho STAT-5. Levels of STAT-5 were revealed with STAT-5 antibody. (*b*) Levels of phospho-STAT-5 were determined by densitometry and the ratio to total STAT-5 calculated. The results are shown as densities relative to IL-2-only treated cells. Data are representative of three independent experiments. (*c*) HT-2 cells were treated with reducing conditions as indicated, lysed and probed with the 4G10 anti-phoshotyrosine antibody. The reducing conditions that affected CD132 signalling did not have a major effect on the overall levels of phosphorylation.

Cytokine receptors using CD132 signal through Jak–STAT pathways. In the case of IL-2 signalling, binding of IL-2 to the surface receptor complex initiates the recruitment of Jak-3, which phosphorylates STAT-5, resulting in its dimerization and translocation to the nucleus to initiate gene transcription [32]. The level of tyrosine phosphorylation of STAT-5 is therefore a direct readout for the presence of a functional IL-2 receptor complex. In order to test the hypothesis that reduction of CD132 inhibits the ability of the IL-2 receptor complex to bind IL-2 and signal through CD132, we measured levels of tyrosine phosphorylation in STAT-5 (figure 5*a*; quantitation in figure 5*b*). IL-2-dependent HT-2 cells were starved of IL-2 for 18 h to give a background level of STAT-5 tyrosine phosphorylation. The cells were reduced using identical conditions to those used to identify the labile disulfide and then either treated with IL-2 for 30 min or first alkylated with NEM and then treated with IL-2 for 30 min. A third sample of cells was treated with IL-2 without prior reduction and alkylation. Cells treated with IL-2 alone resulted in an increased level of tyrosine phosphorylation in STAT-5. However, cells treated with IL-2 after reduction or reduction and NEM alkylation showed STAT-5 tyrosine phosphorylation levels comparable to the control. This shows that conditions known to reduce the labile disulfide bond in CD132 directly inhibit cytokine signalling through the IL-2 receptor complex. Furthermore, the finding that inhibition occurred with or without NEM alkylation of the free cysteines shows that this disulfide does not reform and restore activity once reduced.

To test whether the decrease in STAT-5 tyrosine phosphorylation after reduction of CD132 is a pathway-specific process and that the reducing agent is not inhibiting all tyrosine phosphorylation in the cell, lysates from non-reduced and reduced HT-2 cells were Western blotted with an anti-phosphotyrosine antibody (figure 5*c*). This showed there was no significant decrease in global phosphorylation upon reduction, and that inhibition of phosphorylation of tyrosines in STAT-5 and cell proliferation is pathway-specific through CD132.

## 4. Discussion

CD132 was identified as having a labile disulfide bond in screens to determine the molecular modification of membrane proteins that occurred during inflammation, where it is known that there are changes in the extracellular redox environment both in terms of the secretion of enzymes that can modify disulfide bonds and the overall redox potential [20]. The specific disulfide in CD132 was identified using a variety of mild reducing agents including PDI, TRX and GILT. CD132 was also one of many proteins identified in a model of inflammation. The detailed structural knowledge of CD132 in structures of the receptors and their complexes with IL-2 and IL-4 allows the molecular consequences to be examined [24,26,33]. The Cys^183^–Cys^232^ of CD132 that we identify as labile is solvent exposed in the equivalent structure for the human proteins at the interface of CD132 and IL-2 or IL-4. This disulfide bond bridges two loops in domain 1 of CD132. As described by Wang *et al*., ‘this disulfide bond fixes the bent conformation of loop FG2, where the main chain atoms from residues Ser-207 to Pro-211 directly contact the methylene side chain of residue Gln-126 in IL-2 or Arg-121 in IL-4’ (page 48 of [22]). Thus, reduction of this disulfide probably imparts added flexibility into the loops affecting IL-2 and IL-4 binding signalling (figure 2). Mutation of these Cys residues severely disrupts IL-2 binding to CD132, as shown by cell surface binding of IL-2 to COS-7 cells transfected with CD132 mutants, and surface plasmon resonance analysis on recombinant IL-2 and receptor chains [30,34]. Mutations in several residues at the CD132–cytokine interface, including this Cys^160^–Cys^209^ pair, lead to X-linked severe combined immunodeficiency syndrome (X-SCID) [24,35,36]. Our functional data show that mild reduction inhibits proliferation of an IL-2-dependent T cell clone and the STAT-5 pathway. This complements analysis showing that mutation of either of the Cys residues involved in this disulfide bond inhibits IL-2 binding to its receptor.

CD132 is part of the receptor for several other cytokines (IL-4, IL-21, IL-7, IL-9, IL-15) [32]. There are clear data for the IL-4 receptor showing that the labile disulfide in CD132 is at the interface with the cytokine, and it seems likely that the other cytokines bind in a similar way [22], with data indicating at least some overlap between the sites on CD132 for IL-4 and IL-21 [37]. For IL-4, there is the added complication that IL-4 itself has a labile disulfide-affecting function [13]. Thus, the activity of several cytokine receptors may be affected by a ‘redox regulator’ resulting from changes in the redox potential and the availability of thiol-modifying enzymes such as PDI.

The changes in the redox environment are presumably transient. What happens when conditions return to normal? The effect of reduction of the receptor is reversible over a period of hours; this could either be due to the reduced disulfide bonds reforming after reducing conditions have been removed, or the cells could be turning over the receptor at the cell surface and replacing reduced CD132 with newly synthesized non-reduced CD132. The finding that little spontaneous reforming of active CD132 occurred over 30 min suggests little reformation of the disulfide bond and that receptor turnover is the primary cause of restoration of IL-2-dependent cell proliferation. Presumably, the Cys^183^–Cys^232^ disulfide bond is formed during biosynthesis in the presence of chaperones and restrains the loops.

As CD132 is common to a number of cytokine receptors, there may be a previously undiscovered mechanism controlling the levels of responses of cells to exogenous cytokines. These changes may also occur in other cytokine receptors. From these data, we would predict that disulfides that are solvent-exposed and at the interface with the cytokine may be affected by redox changes with functional effects. Exposed disulfide bonds are not common in other cytokine receptors where there are structural data; for example, none of the disulfides in the interleukin-28 receptor subunit alpha (or interferon-lambda receptor 1; PDB; 3OG6) is exposed. The tumour necrosis factor (TNF) receptor family members are characterized by a high content of disulfide bonds, but in the TNF–TNFR2 structure (PDB; 3ALQ) two Cys residues are in contact with the ligand (TNF), but their partners are solvent-inaccessible. However, there are data to show that TRX 1 can reduce disulfide bonds in the TNF receptor family member CD30 giving altered cytokine binding [38]. This study showed that other TNF receptor family members were not modified, pointing to selected cytokine receptors being affected. Thus, the activity of some cytokines may be moderated in immune responses by changes in redox potential modulated by the release of enzymes such as PDI. Therefore simple quantitation of the expression of a cytokine receptor may not be fully predictive of its ability to signal.

## 5. Material and methods

### 5.1. Labelling of labile disulfide bonds in 2B4 hybridoma cells and spleen cells

The labelling method and identification of proteins by mass spectrometry is given in detail by Metcalfe *et al*. [20]. In brief, 2B4 cells were treated with 2.5 mM Methyl-PEO_12_-maleimide (MPM) to block any free Cys residues, washed and then incubated with the various reducing agents for 30 min at room temperature. The cells were washed and reacted with 2.5 mM MPB, and left to rotate at 4° for 30 min to biotinylate any free sulfydryls formed in the reduction step. After washing, the cells were lysed in 1 per cent Triton X-100 with 5 mM N-ethylmaleimide and membrane glycoproteins purified by lentil lectin affinity chromatography. The modified proteins were repurified using monomeric avidin beads (Pierce Chemical Company, Rockford, IL) and the eluent was digested with PNGase F and trypsin. The tryptic peptide samples were desalted on a C18 packed pipette tip system and injected onto an Ultimate 3000 nano HPLC (Dionex) system coupled with Orbitrap XL mass spectrometer (Thermo Electron).

LPS-induced inflammation was induced in a mouse as described [20]. One microgram of LPS (Sigma Chemical Company, St Louis, MO) in PBS was given intraperitoneally to each adult Balb/c mouse and the spleen taken after 3 h. Control mice received PBS alone. The spleen cells were teased out into RPMI containing 2.5 mM MBP and gently agitated at 4°C for 30 min. The cells were washed and processed as for the cell lines.

### 5.2. Data processing

The data were submitted to the in-house Central Proteomics Facilities Pipeline (CPFP) [39] and the datasets were searched with variable peptide modifications including carbamidomethyl cysteine, oxidized methionine, deamidated asparagine/glutamine, and hydrolysed and non-hydrolysed versions of the appropriate cysteine-modifying label (NEM, MPM or MPB). The resulting peptide identifications from each search engine were validated with PeptideProphet and ProteinProphet and lists compiled at the peptide and protein level [40]. iProphet was used to combine the identifications from three search engines and further refine identifications and probabilities [41]. Normalized spectral index quantitation (SINQ) was applied to the grouped meta-searches to give protein-level quantitation between reduced samples and controls [42]. All lists of peptide and protein identifications were generated with a probability cut-off corresponding to a 1 per cent false discovery rate (FDR) relative to a target decoy database. Quantitated datasets were uploaded to ProteinCenter (Proxion, Thermofisher) for analysis. The dataset was reduced by removing single-peptide identifications and proteins of interest (cell surface, secreted and extracellular) were mined using gene ontology (GO) flags. Resultant protein and peptide lists were exported as tables.

### 5.3. ^3^H-thymidine incorporation by HT-2 cells

HT-2 cells (ATCC; code CRL-1841) were grown in the presence of IL-2 (approx. 100 U ml^−1^ of recombinant rat IL-2 produced in house from a Chinese hamster ovary cell expression system) and then starved of exogenous IL-2 for 18 h. Cells were either reduced with 2.5 mM TCEP or 0.5 μg ml^−1^ TRX (with 5 μM DTT present) in PBS-containing 1 per cent BSA and, if required, alkylated with 2.5 mM MPM for 30 min to alkylate any labile disulfides. A total of 1.5 × 10^4^ cells per well were incubated for 18 h with ^3^H-thymidine in RPMI containing 10 per cent FCS and 2 per cent recombinant rat IL-2 supernatant. Cells were harvested onto filter mats that were saturated in scintillation fluid and radioactivity measured on a scintillation counter. Twelve identical wells were counted for each condition and the experiments were carried out in triplicate.

### 5.4. Reduction and alkylation of IL-2

Recombinant rat IL-2 (Peprotech, Rocky Hill, NJ) was reduced for 30 min with 2.5 mM TCEP in PBS at room temperature. Any labile disulfide bonds formed were alkylated with 2.5 mM NEM in PBS for 30 min. After washing through a micro concentrator (Millipore, YM-10), the IL-2 was denatured (8 M urea, 1 h), reduced (10 mM DTT, 37°C, 30 min) and alkylated (10 mM IAA, 1 h). The IL-2 was thoroughly washed with 25 mM ammonium bicarbonate and then subjected to trypsin digest and mass spectrometry as described previously.

### 5.5. Analysis of STAT-5 phosphorylation state

HT-2 cells were grown in the presence of IL-2 and then starved of exogenous IL-2 for 18 h. A total of 5 × 10^5^ cells were either reduced (2.5 mM TCEP for 30 min) or reduced and alkylated (2.5 mM TCEP + 2.5 mM MPM for 30 min) and then treated with IL-2 (recombinant rat supernatant) for 30 min. Control samples (5 × 10^5^ cells) were treated with IL-2 for 30 min without prior reduction and alkylation. Cells were then lysed in 1 per cent Triton-X100 in PBS and subjected to Western blot analysis with rabbit anti-mouse STAT-5 (3H7, Cell Signaling Technology, Boston, MA) and rabbit anti-mouse phospho (Y694) STAT-5 (Cell Signaling Technology). Densitometry quantitation of the STAT-5 and phosphoSTAT-5 bands was performed using ImageJ (NIH, USA). All experiments were carried out in triplicate.
